# Metagenomic next-generation sequencing as a diagnostic tool in the clinical routine of an infectious diseases department: a retrospective cohort study

**DOI:** 10.1007/s15010-024-02300-2

**Published:** 2024-05-22

**Authors:** Sven Kalbitz, Jörg Ermisch, Nils Kellner, Olaf Nickel, Stephan Borte, Kathrin Marx, Christoph Lübbert

**Affiliations:** 1 Department of Infectious Diseases and Tropical Medicine, Hospital St. Georg, Leipzig, Germany; 2 Department of Laboratory Medicine, Hospital St. Georg, Leipzig, Germany; 3 Jeffrey Modell Diagnostic and Research Center for Primary Immunodeficiency Diseases, ImmunoDeficiencyCenter Leipzig (IDCL) at Hospital St. Georg Leipzig, Leipzig, Germany; 4 Hospital Pharmacy, Hospital St. Georg, Leipzig, Germany; 5 Division of Infectious Diseases and Tropical Medicine, Department of Medicine I, Leipzig University Medical Center, Liebigstr. 20, 04103 Leipzig, Germany; 6 Interdisciplinary Center for Infectious Diseases (ZINF), Leipzig University Medical Center, Leipzig, Germany

**Keywords:** mNGS, Diagnostics, Infectious diseases, Immunodeficiency, HIV infection

## Abstract

**Background:**

Metagenomic next-generation sequencing (mNGS) of circulating cell-free DNA from plasma is a hypothesis-independent broadband diagnostic method for identification of potential pathogens. So far, it has only been investigated in special risk populations (e.g. patients with neutropenic fever).

**Purpose:**

To investigate the extent to which mNGS (DISQVER® platform) can be used in routine clinical practice.

**Methods:**

We collected whole blood specimens for mNGS testing, blood cultures (BC), and pathogen-specific PCR diagnostics. Clinical data and pathogen diagnostics were retrospectively reviewed by an infectious disease expert panel regarding the adjustment of anti-infective therapy.

**Results:**

In 55 selected patients (median age 53 years, 67% male) with heterogeneous diagnoses, a total of 66 different microorganisms and viruses were detected using mNGS (51% viruses, 38% bacteria, 8% fungi, 3% parasites). The overall positivity rate of mNGS was 53% (29/55). Fifty-two out of 66 (79%) potential pathogens detected by mNGS were found in patients with primary or secondary immunodeficiency. The concordance rates of BC and pathogen-specific PCR diagnostics with mNGS testing were 14% (4/28) and 36% (10/28), respectively (p < 0.001). An additional bacterial pathogen (*Streptococcus agalactiae*) could only be detected by BC. Therapeutic consequences regarding anti-infective therapy were drawn from 23 pathogens (35% of detections), with 18 of these detections occurring in patients with immunodeficiency.

**Conclusions:**

We conclude that mNGS is a useful diagnostic tool, but should only be performed selectively in addition to routine diagnostics of infectious diseases. The limited number of patients and the retrospective study design do not allow any further conclusions.

## Introduction

Patients with complex infectious diseases (especially when undergoing anti-infective prophylaxis) pose a challenge even with modern diagnostic laboratory methods, especially when direct pathogen detection is required within a short period of time. In this context, molecular genetic diagnostic procedures are increasingly replacing the established culture-based methods in microbiological diagnostics, even in resource-limited settings [1].

Recent advances in next-generation sequencing (NGS) have laid the foundation for modern investigations into the composition of microbial communities. The use of these NGS methods enables detection and identification of difficult-to-cultivate microorganisms using a culture-independent strategy [2]. In non-infectious diseases, the use of NGS panels, for example in patients with myeloid neoplasms, has already been shown to improve the genetic characterization of the disease compared to conventional methods and demonstrate their clinical utility in routine clinical investigation, leading to better tailored treatments for each patient and potentially better outcomes [3].

Metagenomic next-generation sequencing (mNGS) is a new and rapid method for detecting minute amounts of cell-free pathogen DNA in blood and possibly other media [4]. These DNA fragments are compared with a library of more than 16,000 microbial species and can be clearly assigned, enabling hypothesis-free analysis [5]. Pathogen DNA can be amplified where cultural detection was previously impossible or very difficult. So far, this technology has only been used in pilot studies and in small series of high-risk patients, e.g. hematologic cancer patient, patients with septic shock or critically ill patients with suspected secondary bloodstream infections [5–7]. A major methodological limitation is the lack of detection of RNA viruses.

The purpose of this study was to investigate the extent to which mNGS can be used in routine clinical practice for suspected infections in comparison to other standard diagnostic procedures, in particular blood cultures (BC) and pathogen-specific PCR, in the setting of a tertiary-care hospital.

## Methods

### Setting

The Hospital St. Georg in Leipzig, Saxony, Germany is a large tertiary-care hospital with more than 700 beds and 25 different specialist areas and clinics embedded in the structure of a modern academic teaching hospital. Patients with infectious diseases are primarily cared for in two infectious disease wards, with a total of 44 beds, if intensive care unit (ICU) therapy is not required.

### Study design and patients

This retrospective cohort study was conducted from July 1, 2022 to February 28, 2023. Patients with sepsis, fever of unknown origin (FUO), fever during cytostatic chemotherapy, suspected prosthesis infections, transplant recipients, HIV-infected patients, patients with autoimmune diseases, and patients with primary immunodeficiency were in particular eligible for mNGS testing. Inclusion criteria comprised hospitalization with suspected infection in the hospital's two infectious disease wards or another specialist department with consultative support from the department of infectious diseases and age ≥ 18 years. The indication for the use of mNGS was made by the attending physician after consultation with an infectious disease specialist. Blood samples could be collected at any time when BC or other targeted diagnostics for systemic infections were indicated. Exclusion criteria included participation in any other clinical study, pregnancy and breastfeeding.

### Metagenomic next-generation sequencing (mNGS)

Blood samples were drawn into Streck blood collection tubes and shipped at ambient temperature by a medical logistics integrator to the laboratories of Noscendo GmbH in Reutlingen, Germany. Plasma separation and aliquotation, nucleic acid isolation, quality controls, and library preparation were carried out as previously described [5]. Adequate positive and negative controls accompanied all laboratory and sequencing procedures. Raw sequencing data were subjected to various quality controls comprising Phred score filtering, adapter trimming, complexity filtering, as well as k-mer-based contamination screening, as described previously [5]. To pass the quality filter, read quality needed to surpass a Phred score of 20 and achieve a minimal length of 50 base pairs after quality control. All data generated were analyzed using Noscendo’s DISQVER® platform, as described previously [5]. The DISQVER® platform comprises a curated microbial genome reference database of over 16,000 microbial species covering more than 1,500 pathogens and can detect bacteria, DNA viruses, fungi, and parasites while differentiating contamination and commensals from infective agents. The mNGS report is usually available within 48 h of the sample being sent to the laboratory.

### Blood cultures (BC)

All BC bottles were processed by standard procedures using the BACT/ALERT® VIRTUO® automatic BC detection system (bioMérieux, Marcy-l'Étoile, France), as described previously [8]. Subsequent identification of cultured microorganisms was performed by routine microbiological procedures including matrix-assisted laser desorption ionization time-of-flight (MALDI-TOF) mass spectrometry identification (VITEK® MS; bioMérieux, Marcy-l'Étoile, France).

### Virology

Additional diagnostic tests for viruses from blood samples were only conducted if a viral infection was clinically suspected by the attending physician and consultation with the infectious disease consultant had taken place. The routine virus detection panel for blood samples was performed by quantitative real-time PCR, as per institutional protocol, and included human immunodeficiency virus (HIV), herpes simplex virus type 1 and 2 (HSV-1/2), Epstein-Barr virus (EBV), and human cytomegalovirus (CMV).

### Data review

A panel composed of two senior infectious disease physicians and one microbiology specialist examined medical files, including clinical parameters, previous course of the disease, anti-infective treatments, and results from pathogen diagnostics. Results of the mNGS analysis were assessed for clinical relevance and categorized as to whether the results provided any additional or unique findings or affected anti-infective therapy. Identification of typical BC contaminants, such as coagulase-negative staphylococci (CNS), was assessed for clinical relevance. Contamination was assumed if the suspected isolates were considered as such, either according to clinical documentation or were present in only one BC and no further action was taken.

### Statistical analysis

Statistical analysis was performed using R statistical software (version 4.3.1). Continuous data are presented as the median and interquartile range, categorical data are presented as counts and percentages. Quantitative variables were compared using the Mann–Whitney U test, qualitative variables were compared using the Chi-square test or Fisher’s exact test, as appropriate. A p-value < 0.05 was considered statistically significant.

## Ethics approval

The study was conducted in accordance with the ethical guidelines of the 1964 Declaration of Helsinki and its later amendments and was approved by the local ethics committee (Saxonian Board of Physicians, Dresden, Germany, vote EK-BR-80/23–1).

## Results

### Patients

55 patients (67% male) with a median age of 53 years (range 18 to 84 years) were examined. The majority of patients (43/55; 78%) were treated in medical wards, with 34/55 (62%) being treated in two infectious disease wards. Twenty-three patients (42%) were classified as immunocompromised. Eight patients had HIV infection (CDC stage C3), 7 patients had hematologic cancer diagnoses, 4 patients were transplant recipients, and 4 patients had other primary or secondary immunodeficiencies. Detailed clinical baseline characteristics are shown in Table 1.

**Table 1:** Baseline characteristics of patients

	Patients with immunodeficiency	Patients without immunodeficiency	*P* value	Overall
Number of patients (n)	23	32		55
Age (years)	55 [40.5, 65.0]	52 [38.8, 67.3]	0.844	53 [38.8, 67.3]
Male sex (%)	17 (74)	20 (62)	0.550	37 (67)
Length of hospital stay (days)	25 [18.5, 42.0]	18.5 [9.8, 48.0]	0.339	22 [11.5, 44.5]
Case-mix index (CMI)	3.28 [1.40, 5.43]	1.27 [0.63, 2.90]	0.019	1.82 [1.06, 4.79]
Community-acquired infection (%)	18 (78)	26 (81)	1.000	44 (80)
Intensive care unit (ICU) stay (%)	5 (22)	7 (22)	1.000	12 (22)
In-hospital death (%)	6 (26)	3 (9)	0.199	9 (16)
Charlson comorbidity index (CCI)	4 [3, 6]	1 [0, 2]	< 0.001	2 [1, 4]
Comorbidities (%)				
Hypertension	12 (52)	13 (41)	0.566	25 (45)
Cardiovascular disease	2 (9)	3 (9)	1.000	5 (9)
Pulmonary disease	2 (9)	2 (6)	1.000	4 (7)
Renal disease	14 (61)	8 (25)	0.016	22 (40)
Liver disease	3 (13)	2 (6)	0.697	5 (9)
Diabetes mellitus	5 (22)	4 (12)	0.586	9 (16)
Positive diagnostic results				
mNGS	20 (87)	9 (28)	< 0.001	29 (53)
BC	5 (22)	4 (12)	0.586	9 (16)
Pathogen-specific PCR	11 (48)	3 (9)	0.001	14 (25)

*BC *blood culture(s), *mNGS *metagenomic next-generation sequencing, *PCR *polymerase chain reaction

Demographic data are presented as the median and interquartile range (IQR) or as counts and percentages, as appropriate

### Microorganisms and viruses detected

Sixty-six different potential pathogens were detected by mNGS in 29 out of 55 patients (53%), with a proportion of 51% viruses, 38% bacteria, 8% fungi, and 3% parasites (Table 2).

**Table 2:** Microorganisms and viruses detected by mNGS

	Patients with immunodeficiency (*n* = 23)	Patients without immunodeficiency (*n* = 32)	Overall
Number of microorganisms and viruses (%)	52 (79)	14 (21)	66
Bacteria (%)	17 (33)	8 (57)	25 (38)
*Escherichia coli*	4	0	4
*Enterococcus faecium*	3	0	3
*Klebsiella pneumoniae*	1	2	3
*Lactococcus lactis*	1	1	2
*Actinomyces graevenitzii*	1	0	1
*Bacteroides fragilis*	1	0	1
*Burkholderia contaminans*	1	0	1
*Burkholderia multivorans*	1	0	1
*Corynebacterium striatum*	1	0	1
*Mycobacterium avium*	1	0	1
*Pseudomonas aeruginosa*	1	0	1
*Staphylococcus aureus*	1	0	1
*Enterococcus faecalis*	0	1	1
*Staphylococcus epidermidis*	0	1	1
*Staphylococcus hominis*	0	1	1
*Citrobacter koseri*	0	1	1
*Streptococcus pyogenes*	0	1	1
Fungi (%)	5 (10)	0 (0)	5 (8)
*Pneumocystis jirovecii*	2	0	2
*Candida albicans*	1	0	1
*Lichtheimia corymbifera*	1	0	1
*Rhizomucor miehei*	1	0	1
Viruses (%)	28 (54)	6 (43)	34 (51)
*Epstein-Barr virus*	10	3	13
*Human cytomegalovirus (CMV)*	7	1	8
*Human alphaherpesvirus 1 (HSV-1)*	4	0	4
*Human betaherpesvirus 6B (HHV-6B)*	2	0	2
*BK polyomavirus*	1	0	1
*Human mastadenovirus D*	1	0	1
*Human polyomavirus 2*	1	0	1
*Primate bocavirus 2*	1	0	1
*Torque Teno virus 5 (TTV-5)*	1	0	1
*Human betaherpesvirus 6A (HHV-6A)*	0	1	1
*Torque Teno virus 16 (TTV-16)*	0	1	1
Parasites (%)	2 (4)	0 (0)	2 (3)
*Toxoplasma gondii*	1	0	1
*Echinococcus multilocularis*	1	0	1

Data are presented as counts and percentages

*BC *blood culture(s), *mNGS *metagenomic next-generation sequencing, *PCR *polymerase chain reaction

Among 66 potential pathogens detected by mNGS, 34 viruses, 25 bacteria, 5 fungi, and 2 parasites were found. The most frequently detected virus was Epstein-Barr virus in patients with immunodeficiency. Data are presented as counts and percentages.

Metagenomic NGS findings led to clinically relevant additional information in 18 out of 55 cases (33%), mostly in patients with immunodeficiency (13/18; 72%). In the 8 patients with stage C3 HIV infection, ≥ 2 different potential pathogens were detected in each case (28 different pathogens in total). In 33 of the pathogens detected by mNGS, detection was also promptly achieved using BC or targeted PCR diagnostics (10 pathogens by BC, 23 by PCR). In only one case mNGS testing was negative, but BC revealed *Streptococcus agalactiae*; the corresponding mNGS sample was taken 13 h after BC. The concordance rates of BC and targeted PCR diagnostics with mNGS testing were 14% (4/28) and 36% (10/28), respectively (p < 0.001).

### Therapeutic consequences

A therapeutic consequence regarding adjustment of anti-infective therapy was drawn from 23 pathogen detections (35%), with 18 of these detections occurring in patients with immunodeficiency (Table 3).

**Table 3:** Diagnostic contribution and expert evaluation of the 66 microorganisms and viruses detected by mNGS comparing to BC positivity and targeted PCR diagnostics, stratified by patients with and without immunodeficiency

	Number of microorganisms and viruses detected in patients with immunodeficiency	Number of microorganisms and viruses detected in patients without immunodeficiency	*P* value	Overall
mNGS ( +) (%)	52 (79)	14 (21)		66
Concordance with BC				
mNGS ( +)/BC ( +) (%)	7 (14)	3 (21)	0.750	10 (15)
mNGS ( +)/BC (−) (%)	45 (86)	11 (79)	0.750	56 (85)
Concordance with pathogen-specific PCR				
mNGS ( +)/PCR ( +) (%)	21 (40)	2 (14)	0.133	23 (35)
Expert evaluation				
mNGS as relevant additional finding (%)	22 (42)	6 (43)	1.000	28 (42)
Adjustment of anti-infective therapy due to mNGS findings (%)	18 (35)	5 (36)	1.000	23 (35)

## Discussion

Metagenomic NGS offers clinicians a significant extension to the routine diagnosis of pathogens. The direction-independent detection strategy of the method makes it possible to detect a broad spectrum of potential pathogens (with the exception of RNA viruses) within 48 to 72 h [4, 5]. Especially in cases of unclear fever, infections with very rare pathogens or infections in immunocompromised patients with multiple opportunistic pathogens, mNGS offers diagnostic advantages. In particular, added diagnostic value has so far been demonstrated for patients with neutropenic fever [6], critically ill patients with suspected bloodstream infections [7], patients with periprosthetic joint infections [9], and sepsis patients [5, 10]. There is now also evidence of a higher pathogen detection rate with mNGS when combining transbronchial lung biopsy and mNGS in peripheral pulmonary infectious lesions [11] and when screening blood donors, as previously reported from China [12].

Despite the existing selection bias, our study results indicate that patients with primary or secondary immunodeficiency particularly benefit from this type of diagnosis. Only few retrospective studies have so far evaluated mNGS diagnostics in patients with HIV infection [13]. To our knowledge, this is the first study to report on the use of mNGS for the detection of pathogens in HIV-infected patients in routine clinical practice. Hogan et al. also investigated mNGS in a routine setting and were able to demonstrate a therapeutic benefit in a small percentage of cases [14]. In our work, we have succeeded in demonstrating an additional benefit for immunocompromised patients, particularly with regard to pathogens that are difficult to cultivate or are difficult and time-consuming to detect. Quantitatively, more pathogens were detected in patients with immunodeficiency. However, with regard to the clinical consequence in terms of a change in anti-infective therapy, it was shown that a therapeutic consequence was achieved in 29% of cases in both patients with and without immunodeficiency. Nevertheless, it should be noted that in our study all pathogens that are difficult or impossible to cultivate were detected by mNGS in immunodeficient patients (*Pneumocystis jirovecii*, *Echinococcus multilocularis*, *Toxoplasma gondii*, Mucorales). Therefore, mNGS could make a useful contribution particularly in patients with late-stage HIV infection.

Negative results in mNGS testing must always be interpreted in the context of the clinical situation, as cell-free DNA is not detectable in all stages of infection [2]. However, it should be noted that a negative result does not rule out an existing infection with certainty. For this reason, the methods of classical diagnostics should always be carried out in addition if there is a corresponding clinical suspicion. Due to the descriptive design of this study, no statement can be made on the extent to which mNGS is superior to conventional infection diagnostics, nor can a statement be made on the basis of the available data as to whether the cost–benefit analysis is positive. The limited number of patients and the retrospective study design do not allow any further conclusions to be drawn.
